# The Road Less Traveled: A Pathway Leading to Research at a Primarily Undergraduate Institution

**DOI:** 10.1021/acsomega.5c04501

**Published:** 2025-07-21

**Authors:** Timothy D. Lash

**Affiliations:** Department of Chemistry, 6049 Illinois State University, Normal, Illinois 61790-4160, United States

## Abstract

Scholarly activities in primarily undergraduate institutions involve very different challenges from those in major research universities. In this essay, the author discusses how he initiated a research program involving undergraduates at Illinois State University following a number of missteps and false starts. Although undergraduate researchers have much to learn, and the levels of commitment vary considerably, they can nevertheless often make important contributions.

## Graphical abstract

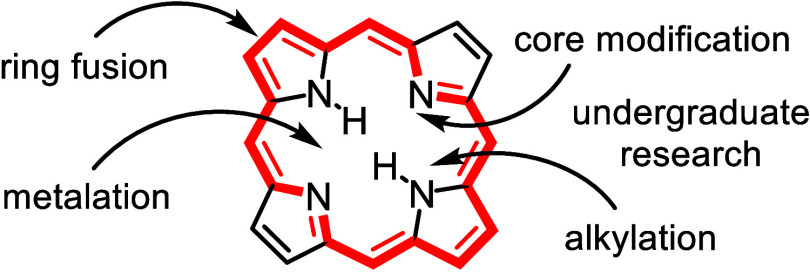

## 1 Introduction

I am always a little disconcerted by young people who have clear goals for their future. That is not to say that this is anything other than laudable, as making good choices at an early point in their lives can provide the foundation for later successes. However, my own decisions at that stage were not well formulated and I had only the vaguest idea of where my career was destined to go. Much of this was due to immaturity and a lack of confidence, and I did not believe then that I was well suited for teaching at the college level. Subsequently, it became my passion. My initial pathway was somewhat chaotic but, as I came to believe in myself and better understand what I wanted to achieve, the road less traveled brought me to where I needed to go.

From time to time, Assistant Professors in 4 year colleges will email me, wanting to know how I developed a successful research program at a non-Ph.D. institution. Unfortunately, I do not have much to tell them as it primarily involved hard work and luck, although formulating a program that is achievable with the resources available is important. Although conducting collaborative research has merit, to my mind it is not a substitute to developing your own ideas.

I received a B.Sc. degree from the University of Exeter in the U.K. and continued on to the University of Wales, College of Cardiff (now Cardiff University) to work on my Ph.D. I elected to work with Professor A.H. Jackson, who was well-known for his studies in the area of indole and porphyrin chemistry. My project involved the synthesis of abnormal porphyrin metabolites associated with porphyria or environmental poisoning (e.g., from hexachlorobenzene). The results provided insights into the heme biosynthetic pathway, a topic that I would return to at a later date. After I completed my Ph.D. dissertation, it took me several years to find myself. In the fall of 1981, I had the opportunity to take a one-year position in the Department of Chemistry at the University of Wisconsin–River Falls (UW-RF) as a sabbatical leave replacement. UW-RF is a primarily undergraduate institution and at that time the Chemistry department had a strong undergraduate program but did not offer graduate degrees. This turned out to be a major turning point in my life. I really had no clear idea of how US colleges operated and the opportunities that I was afforded during that year were both enjoyable and insightful. In addition to teaching classes in organic chemistry, I was able to make some initial steps toward developing porphyrin-related research projects. In particular, we looked into the synthesis of an oxophlorin analogue **1** (Figure 1),^1^ a study that was inspired by previous research conducted by others on oxophlorins and related systems.^2,3^ Around this time, I speculated about the potential synthesis of hydrocarbon analogues **2** of the porphyrins.^4^ The idea was to introduce cyclopentadiene units in place of the usual pyrrole rings. Although our initial efforts quickly stalled, I coined the name quatyrin for this system.^5^ This in turn was based on the suggestion by others that nonconjugated tetrafuran macrocycles **3** can be considered to be oxa-derivatives of the hypothetical ring system quaterene **4** (Figure 1).^6^ It was some time before I could return to this concept but the name quatyrin was eventually introduced into the literature.^5^ Independently, Vogel had discussed this type of structure as a conceptual bridge between porphyrins, porphyrin isomers and heteroporphyrins, initiating groundbreaking research in this area,^7^ but it was some years before “carbaporphyrins” were properly investigated.^8^

**Figure 1:**
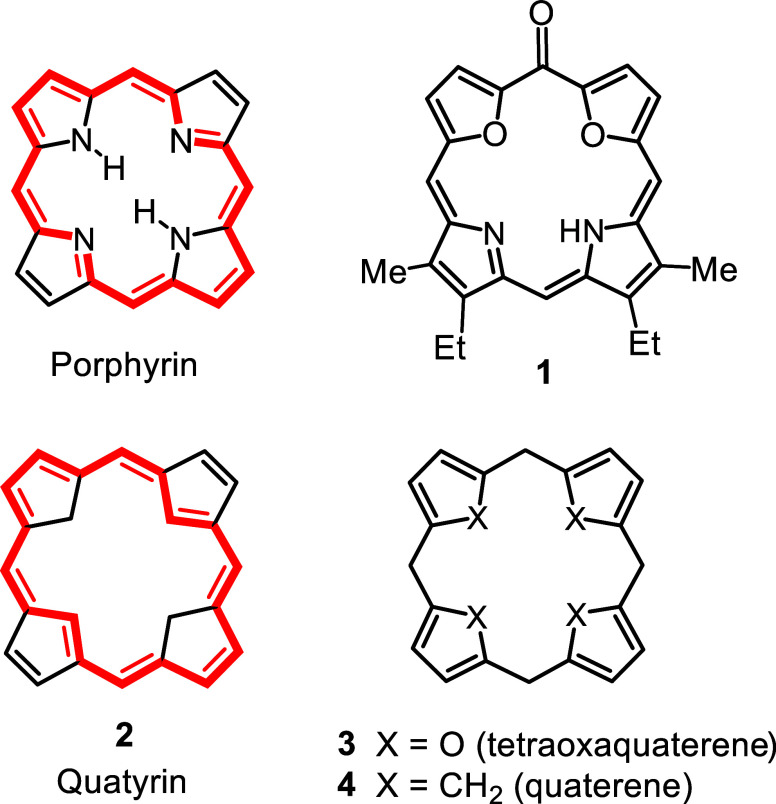
Structures of porphyrin and related systems.

## 2 Discussion

Following my stay in River Falls, I took a tenure track position at a small college in South Dakota. Although I obtained useful teaching experiences, the lack of facilities made it virtually impossible to carry out a worthwhile research program. For that reason, I quickly moved on to take a position in Fall 1984 as an Assistant Professor at Illinois State University (ISU) in Normal, Illinois and in most respects, this is where my career really got started. The Chemistry Department has a small M.S. program but is primarily focused on the undergraduate curriculum. At that time, ISU had a thriving ACS certified Chemistry B.S. degree program, and in fact one year we were ranked fifth in the nation for the number of degrees awarded. Although these numbers have dropped over the years, a trend that is mirrored across the nation, we still maintain a healthy B.S. degree program. The facilities available at ISU in the 1980′s were an improvement over those that I had had access to previously but they were still not a strong as I would have liked. In particular, we had to rely on a 60 MHz CW NMR spectrometer for our early studies, an instrument that was only adequate for the characterization of relatively simple structures and would not facilitate investigations into complex systems. This led me to develop undergraduate research projects that could be achieved within these confines. I was encouraged to a great extent by the success of more established ISU Chemistry faculty who published around 40 papers per year while maintaining substantial teaching responsibilities. I was particularly impressed by the Stevenson group as they regularly published papers in *J*. *Am*. *Chem*. *Soc*., as well as developing a novel methodology for isotope separations that received international recognition.^9^ Their successes were the encouragement that I needed, as they demonstrated the potential of the teacher-scholar model. In my seventh year at ISU. we were able to obtain funding from NSF to purchase a 300 MHz NMR spectrometer and this enabled my research program to become far more productive.

A number of cycloalkanoporphyrins (CAPs **5**–**8**, Figure 2) had been identified in petroleum, oil shales and organic-rich sediments around this time^10^ and these structures were considered to be achievable worthwhile targets for synthesis in my early years at ISU.^11^ It seemed likely that there would be other researchers in the field with superior facilities who would also take an interest in these structures, but we anticipated that they would introduce the fused carbocyclic rings at a late stage in the synthesis after the construction of the porphyrin macrocycle. We elected to take a different approach where the macrocycle was constructed around the carbocyclic ring! This work had numerous challenges, but students were able to prepare many previously unknown heterocyclic structures as the key intermediates by applying a variation of the Knorr pyrrole synthesis (Scheme 1). In fact, in a major review on this methodology 10 citations were made to our work and nearly all of these papers were coauthored by undergraduates.^12^ Pyrroles **9** fused to 5, 6, 7, 8, 9, 10, 12, 15 and 16-membered rings were prepared, and these were successfully applied to the synthesis of numerous CAPs **10** (Scheme 1), including naturally occurring petroporphyrins such as deoxophylloerythroetioporphyrin (DPEP, **5**).

**Figure 2:**
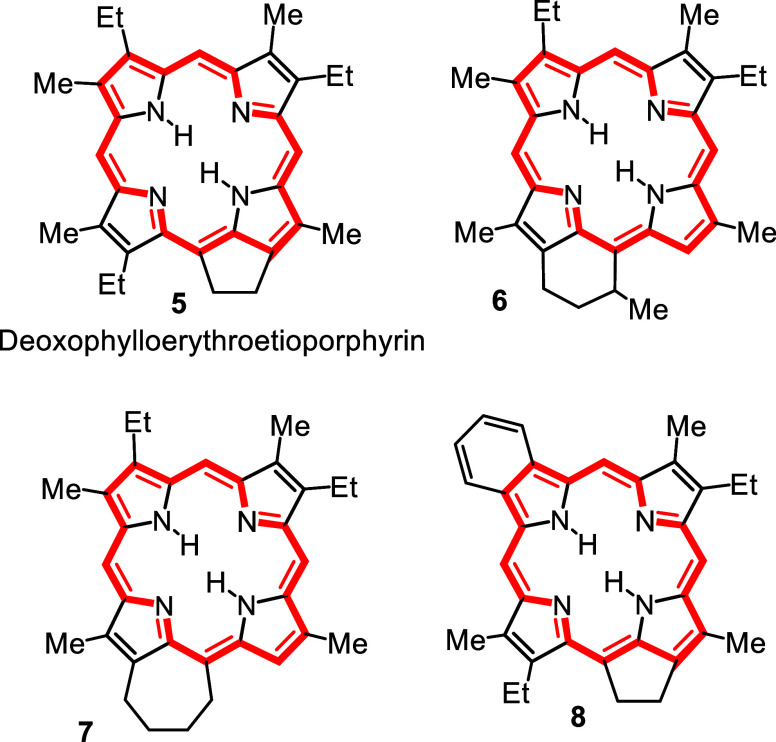
Examples of cycloalkanoporphyrin structures identified in oil shales and petroleum.

**Scheme 1:**
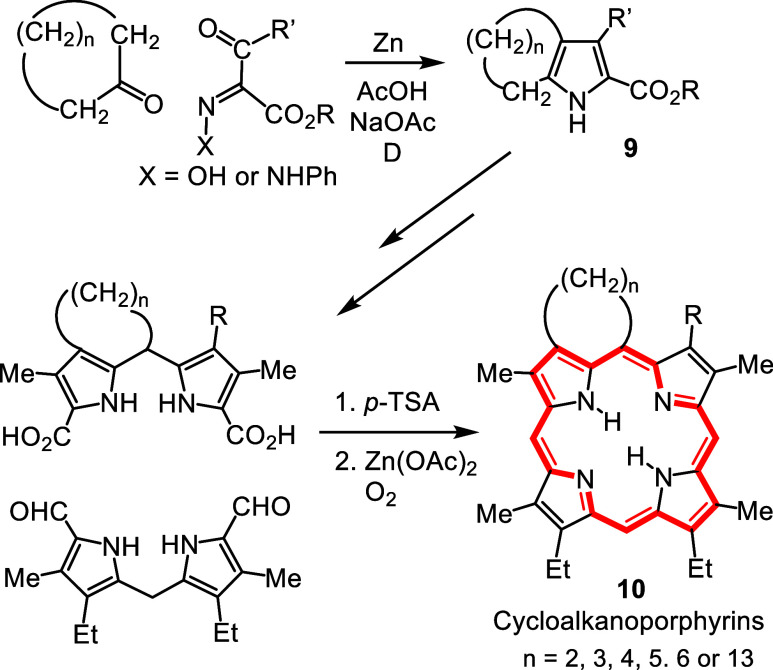
Synthesis of Cycloalkanoporphyrins from Cyclic Ketones

During the course of these studies, we became interested in the synthesis of porphyrins with fused aromatic rings.^13^ Benzoporphyrins (e.g., **8**) have been identified in oil shales and petroleum,^14^ although the origin of these structures is not well understood. It was speculated that naphthoporphyrins might also be present in organic-rich sediments and the synthesis of a naphtho­[1,2-*b*]­porphyrin **11** (Figure 3) was undertaken.^15^ Porphyrins have many applications and have been extensively investigated as photosensitizers in photodynamic therapy (PDT).^16^ As bodily tissues can only be penetrated by light with wavelengths >650 nm, porphyrin-type systems with absorptions in the red or far red have the potential to act as superior photosensitizers in PDT applications. Barton and Zard had reported a valuable synthetic route to pyrrole esters from the reaction of isocyanoacetate esters with nitroalkenes in the presence of a non-nucleophilic base^17^ and we speculated that this methodology might also be applied to the preparation of *c*-annulated pyrroles **12** from nitroaromatic compounds **13** (Scheme 2).^18^ Although this strategy presupposed that the nitroaromatic compounds possessed a degree of nitroalkene character, this approach allowed the synthesis of diverse pyrrolic derivatives.^13,18^ Related studies were also reported by Ono and co-workers.^19^ Our investigations not only provided access to novel pyrrolic derivatives but also afforded suitable precursors for the construction of highly conjugated β,β’-fused porphyrins such as phenanthroporphyrins **14** and acenaphthoporphyrins **15** (Figure 3).^20^ The spectroscopic properties of these porphyrins varied considerably but in some cases strongly red-shifted chromophores were obtained. In a major review on the application of the Barton-Zard reaction published in 2005,^21^ 17 citations to our studies were noted and once again most of the cited papers were coauthored by ISU undergraduate and MS-level graduate students.

**Figure 3:**
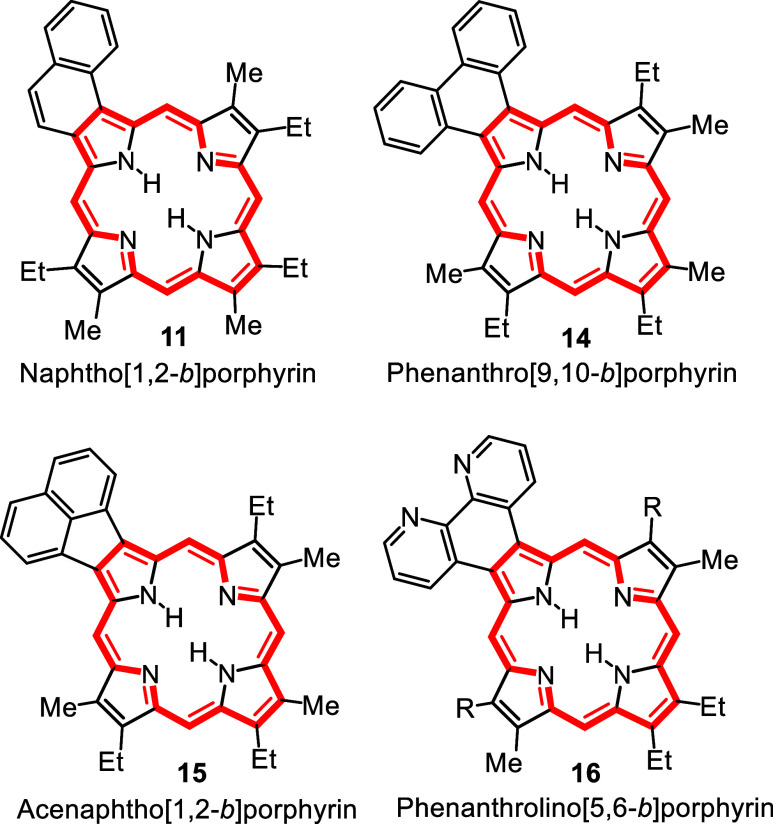
Examples of porphyrins with fused aromatic rings.

**Scheme 2:**
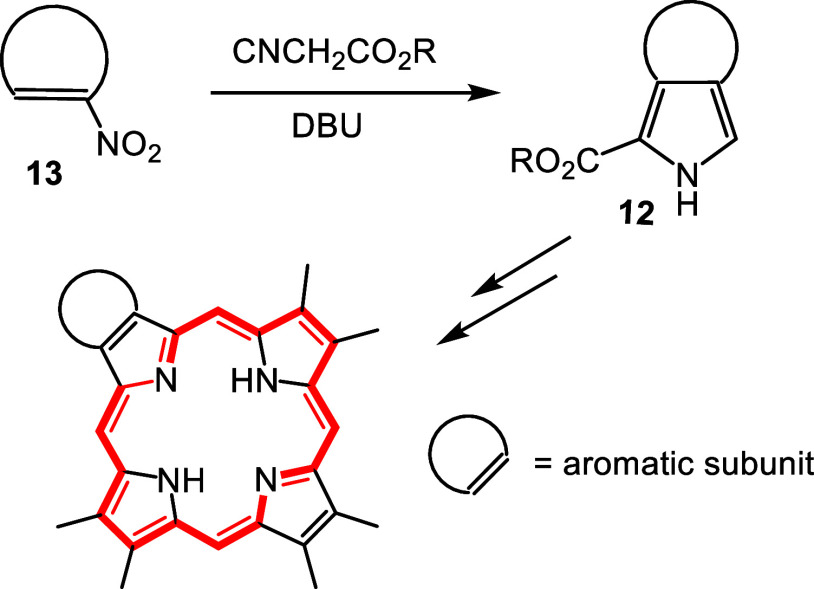
Synthesis of Annulated Porphyrins from Nitroaromatic Compounds

In parallel with this work, my attention returned to intermediates in the heme biosynthetic pathway. The enzyme coproporphyrinogen oxidase found in aerobic organisms converts two of the propionic acid side chains in coproporphyrinogen III into vinyl substituents affording protoporphyrinogen IX, a process that involves both decarboxylation and oxidation (Scheme 3). This important enzyme was poorly understood, both in terms of substrate recognition and mechanism of action. My aim was to probe the enzyme with synthetic analogues of the true intermediates, and I enlisted the help of a biochemistry colleague, Professor Marjorie Jones. Marge Jones has been an amazingly productive researcher and has mentored a large number of undergraduate researchers at ISU. These studies provided a stronger concept of substrate recognition and importantly, allowed us to propose the now generally accepted model for the catalytic activity of aerobic coproporphyrinogen oxidase.^22^ Notably, these projects necessitated collaboration in addition to involving undergraduate students.

**Scheme 3:**
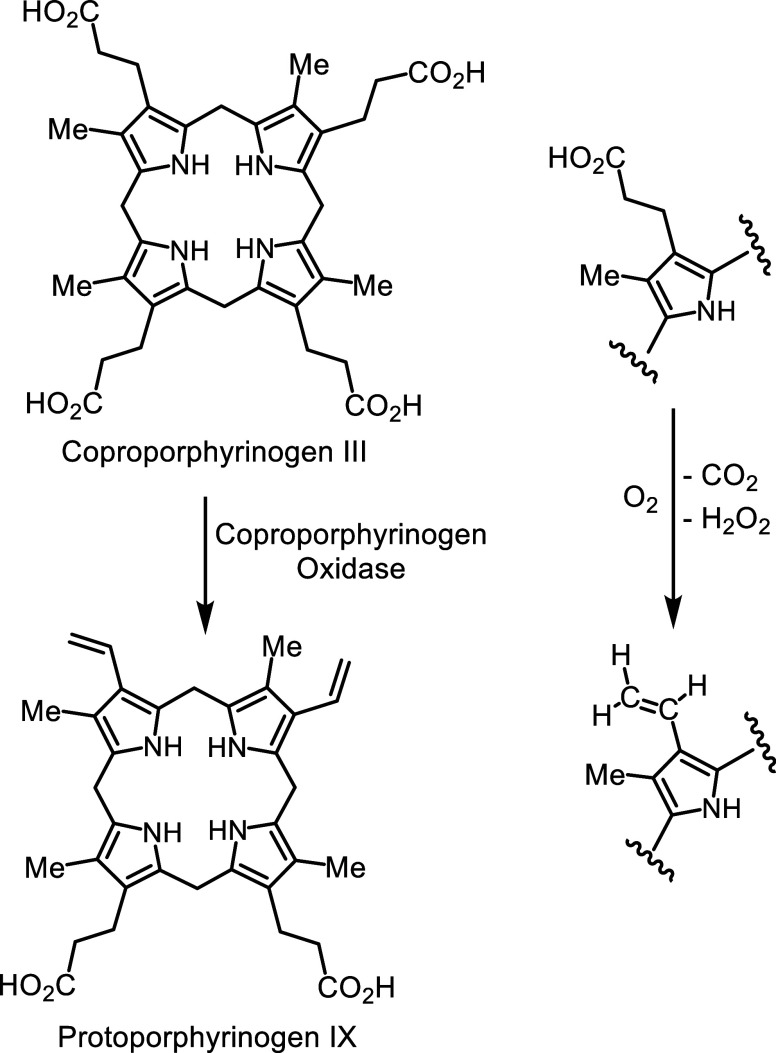
Action of Coproporphyrinogen Oxidase on Coproporphyrinogen III

In our syntheses of exotic porphyrinoid systems, we initially relied on the “2 + 2” MacDonald condensation of dipyrrolic fragments (Scheme 4)^23^ but this failed when we attempted to prepare porphyrins fused to 1,10-phenanthroline units **16** (Scheme 5).^24^ This led to our group adopting a “3 + 1” variant of this strategy.^23^ Although the “3 + 1” approach (Scheme 4) had been used in the early 1970′s by Johnson in the preparation of oxa- and thiaporphyrins,^23^ it did not see further application for nearly 25 years. This was due in part to difficulties in preparing tripyrrane intermediates **17**. However, Sessler reported a straightforward synthesis of a tripyrrane,^25^ and we adapted this methodology using milder acidic conditions to prepare tripyrrolic intermediates for our studies. Although the “2 + 2” route was totally unsuccessful in the synthesis of phenanthroline-fused porphyrins, the ‘3 + 1′ methodology afforded exceptionally high yields of this system (Scheme 5).^24^ Around this time, two groups independently reported the serendipitous isolation of N-confused porphyrins **18**,^26^ a type of porphyrin isomer with an inverted pyrrole ring. Importantly, NCPs replace an internal nitrogen atom with a carbon, and they therefore represent examples of carbaporphyrin-type structures (Figure 4). This system has attracted a considerable amount of attention,^26^ in part due to the ease with which it generates diverse organometallic derivatives. These early reports inspired us to prepare new families of carbaporphyrinoid systems using the “3 + 1” variant on the MacDonald condensation, including oxybenziporphyrins **19**, tropiporphyrins **20**, benzocarbaporphyrins **21** and azuliporphyrins **22** (Scheme 6), all of which were reported in the three years that followed the discovery of NCPs.^8^ These systems demonstrate varying degrees of aromatic character and exhibit unusual reactivity, including regioselective oxidation reactions and the formation of stable organometallic complexes under mild conditions.^8^ Importantly, in collaboration with my ISU colleague Professor Gregory Ferrence, we have characterized many of these systems by X-ray crystallography in addition to using multiple spectroscopic techniques. Studies in this area have dominated our recent efforts and many unique porphyrinoid structures have been investigated. Furthermore, the construction of porphyrin analogues with further core modification, including dicarbaporphyrins, has been achieved and additional modifications to carbaporphyrin structures have been made by core alkylation, ring fusion and metalation. This area of research continues to be very productive and has provided important insights into π-conjugation within macrocyclic systems. Undergraduate collaborators have been involved throughout in the development of this chemistry.

**Figure 4:**
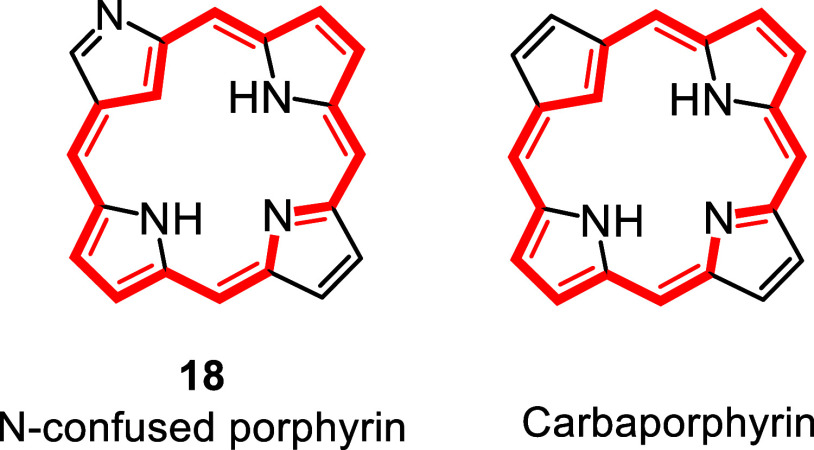
N-confused porphyrin and carbaporphyrin.

**Scheme 4:**
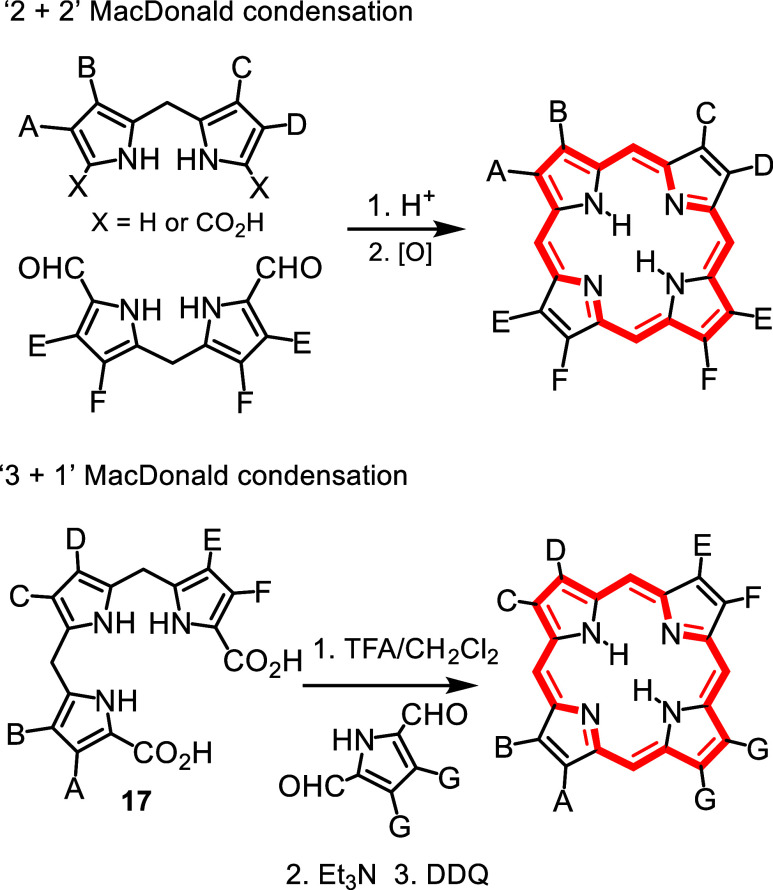
“2 + 2” and “3 + 1” MacDonald Condensations

**Scheme 5:**
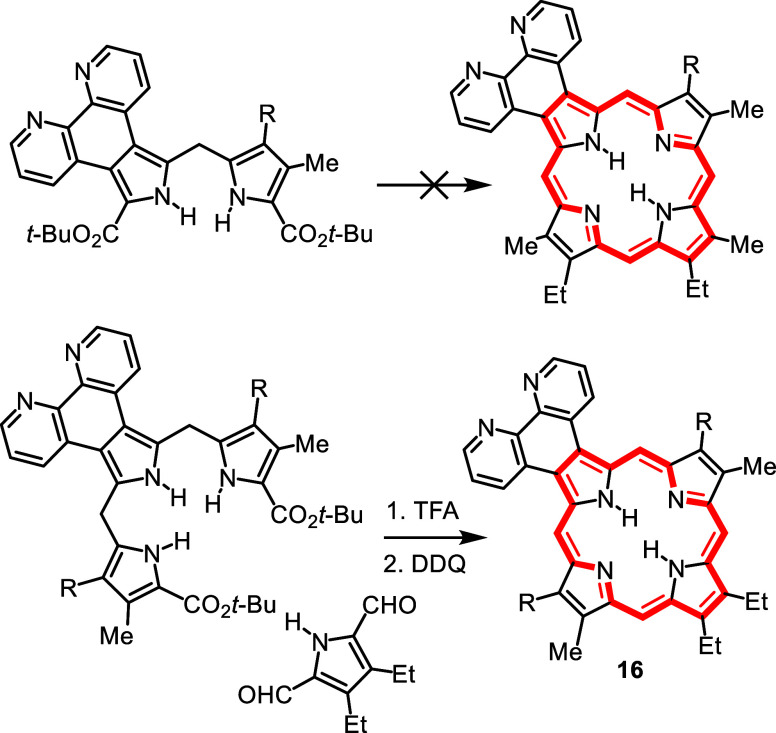
Synthesis of Phenanthrolinoporphyrins

**Scheme 6:**
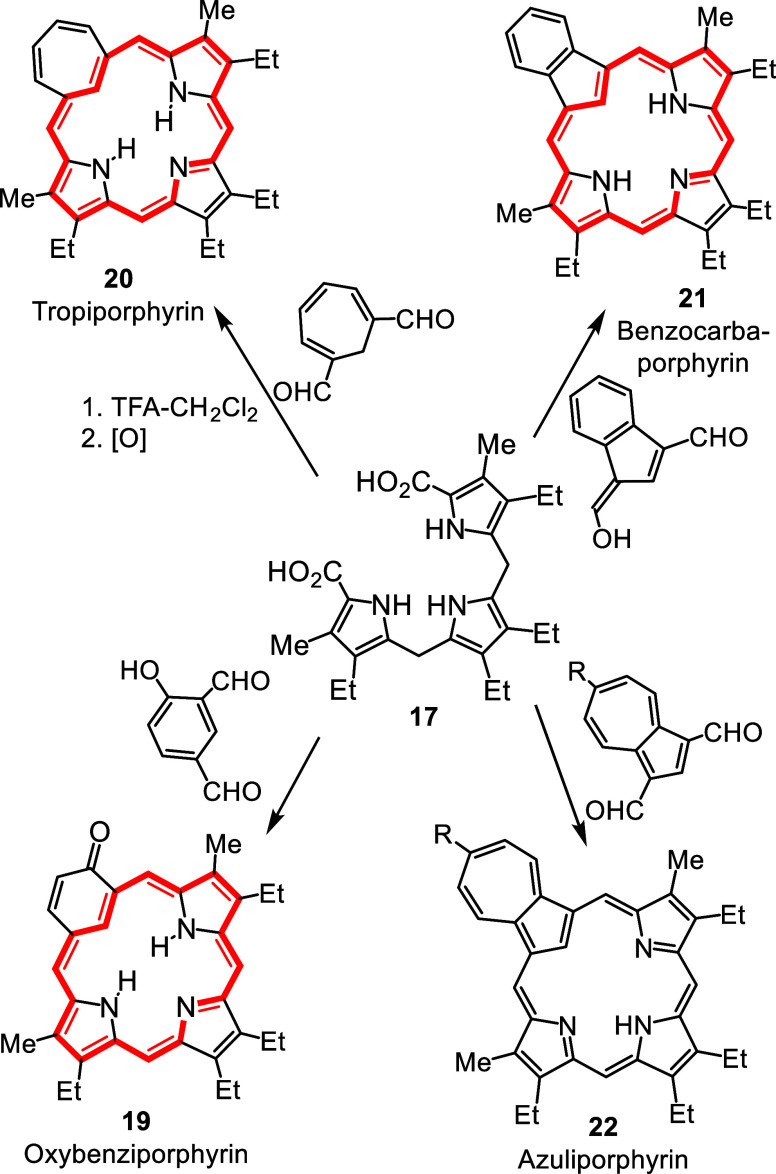
Synthesis of Carbaporphyrinoid Systems

Working with undergraduates is rewarding but can also be very demanding. In addition, projects can take far longer to reach fruition than they would do in a Ph.D. awarding department. It is necessary to work directly with these students to provide the training that they need, and we hold weekly group meetings as well. I should mention that it is very rare for our department to host postdoctoral associates, although MS-level graduate students can help out when I am not available. Anyone seeking to develop an undergraduate research program needs to be willing to send time in the lab, both working alongside the students and in running further experiments. Tenure track faculty at ISU teach many of the organic chemistry lab classes and in some respects, research is a continuation of these efforts. Over the last five years, I completely revised the organic lab manuals that we use, incorporating relatively up to date spectroscopic methods such as DEPT-135 NMR and two-dimensional (2D) techniques such as ^1^H–^1^H COSY and HSQC NMR spectroscopy. This is intended to be accessible to the broad range of students who enroll in these classes while at the same time encouraging chemistry majors to take the senior elective course *Structural Determination in Chemistry*, a class that I also developed which further emphasizes modern spectroscopic methods. Students are motivated by seeing faculty mentors in a lab setting who enthusiastically immerse themselves in experimental studies, and it is important to understand that teaching and research activities are intimately connected.

Funding is also important, and we have been fortunate to receive significant support over the years. Our early work was primarily supported by the ACS Petroleum Research Fund (ACS PRF) and while these grants were not large, they provided the foundations for our later studies. In fact, I am exceedingly grateful to ACS PRF as it would have been difficult to establish my research program without them. Subsequently, we received two NIH AREA grants to conduct our work on coproporphyrinogen oxidase and ten NSF grants under the Research in Undergraduate Institutions (RUI) program. Some additional support was obtained from the Dreyfus Foundation, and undergraduates in my group have received scholarships from the Beckman and Goldwater Foundations and industrial awards from Abbott, Baxter, Johnson and Johnson, Pfizer, and Allied Signals. These grants and scholarships enabled us to develop a competitive program that has resulted in over 240 publications, and 90 different undergraduates have been coauthors on approximately 50% of these papers. These students have diverse backgrounds (Figure 5), and their goals are also extremely varied. While some take positions in industry, others pursue graduate degrees in Chemistry or move on to medical school, dentistry, pharmacy and physician assistant (PA) programs. In addition to contributing to publications in major international journals, undergraduates are encouraged to present their work at regional or national meeting. In recent years, this has commonly been at National ACS meetings (Figure 6). The costs are covered in part by funding from our ChemClub, which makes a profit from selling lab manuals, and from small grants provided by ISU. The remaining expenses are taken from my NSF grants. These activities encourage active participation in research, and this greatly enhances the experience.

**Figure 5:**
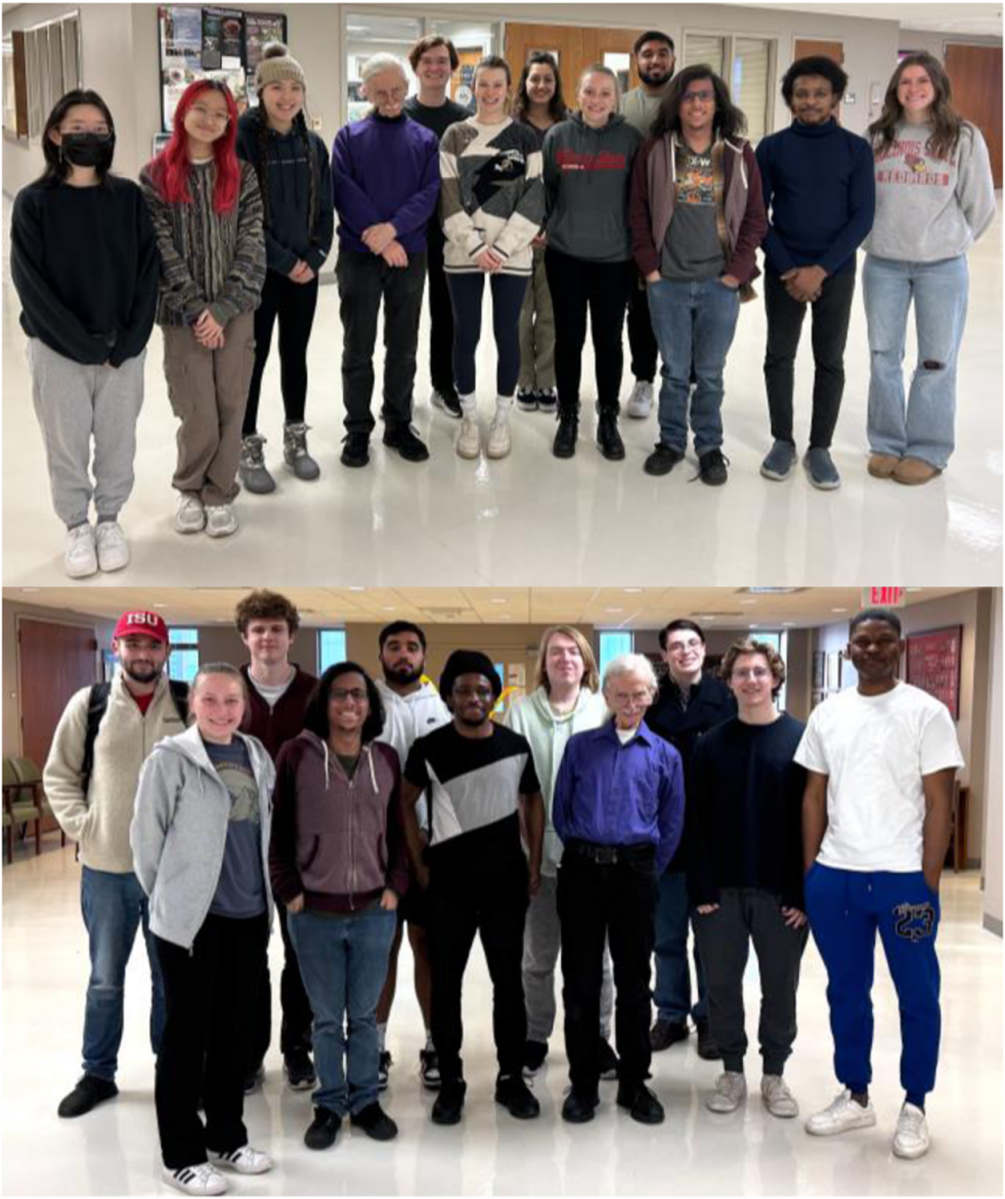
Lash research group Fall 2023 (top) and Spring 2025 (bottom) showing the diversity of undergraduate researchers.

**Figure 6:**
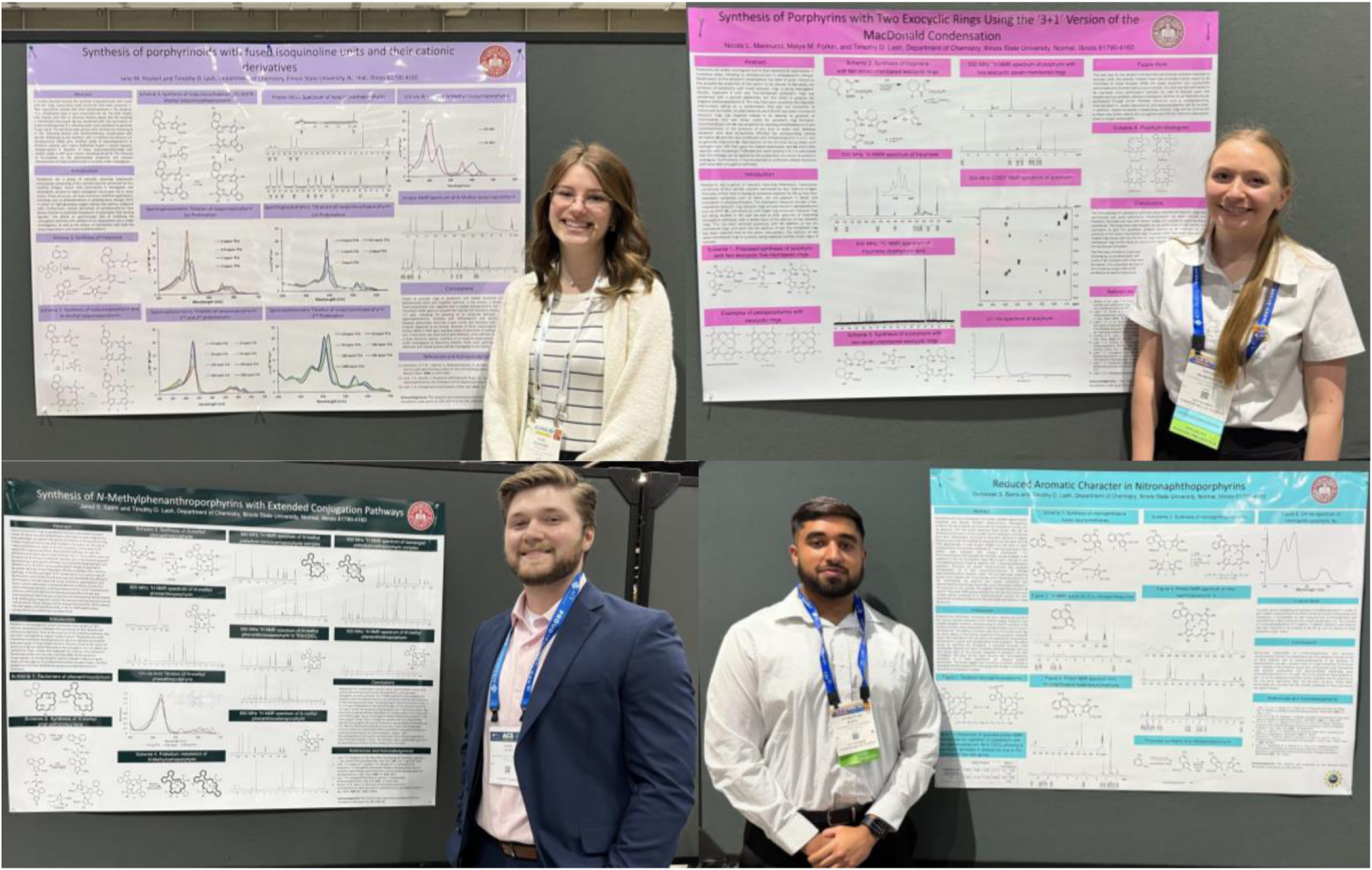
Selected undergraduate poster presentations at recent National ACS Meetings. Clockwise from top left: Jane Hostert (New Orleans, Spring 2024), Nicole Marinucci (San Diego, Spring 2025), Gursewak Bains (San Diego, Spring 2025) and Jared Salrin (Indianapolis, Spring 2023).

Fifteen years ago, I was treated for late-stage oral cancer and could not work full time for most of the following year. However, I was able to come into the department virtually every day and continue working with my students. I believe that the continuation of these activities was beneficial to my recovery and allowed us to maintain a worthwhile research program in the ensuing years.

There is no one way to run a research program. Faculty have different personalities, strengths and weaknesses, and many different approaches can be successful. However, it is necessary to set realistic achievable goals. These must take the available facilities into account as well as the limited amount of time that undergraduate researchers can spend on their projects. However, the research should also be worthwhile and competitive, and it needs to excite the interest of the students. In some cases, undergraduate research provides a bridge to graduate school but for those that move in a different direction it should be a positive experience.

## 3 Conclusions

A career dedicated to teaching and research at a primarily undergraduate college or university is challenging but can be very rewarding. It requires dedication and a genuine passion for both activities and can lead to scholarly outcomes that are internationally competitive. Students gain experience and direction from carrying out undergraduate research and it is to be hoped that these types of activities will be encouraged in the future.
